# A Cloud-Based Platform for Harmonized COVID-19 Data: Design and Implementation of the Rapid Acceleration of Diagnostics (RADx) Data Hub

**DOI:** 10.2196/72677

**Published:** 2025-08-20

**Authors:** Marcos Martínez-Romero, Matthew Horridge, Nilesh Mistry, Aubrie Weyhmiller, Jimmy K Yu, Alissa Fujimoto, Aria Henry, Martin J O'Connor, Ashley Sier, Stephanie Suber, Mete U Akdogan, Yan Cao, Somu Valliappan, Joanna O Mieczkowska, Ashok Krishnamurthy, Michael A Keller, Mark A Musen, RADx Data Hub Team

**Affiliations:** 1 Stanford University Stanford Center for Biomedical Informatics Research Palo Alto, CA United States; 2 Booz Allen Hamilton Inc. McLean, VA United States; 3 University of North Carolina at Chapel Hill Renaissance Computing Institute (RENCI) Chapel Hill, NC United States; 4 see Authors' Contributions section

**Keywords:** COVID-19 surveillance, public health data infrastructure, data harmonization and integration, health disparities, FAIR data sharing, cloud-based data platform, pandemic response informatics, secondary data analysis, metadata standards, digital health research

## Abstract

**Background:**

The COVID-19 pandemic exposed significant limitations in existing data infrastructure, particularly the lack of systems for rapidly collecting, integrating, and analyzing data to support timely and evidence-based public health responses. These shortcomings hampered efforts to conduct comprehensive analyses and make rapid, data-driven decisions in response to emerging threats. To overcome these challenges, the US National Institutes of Health launched the Rapid Acceleration of Diagnostics (RADx) initiative. A key component of this initiative is the RADx Data Hub—a centralized, cloud-based platform designed to support data sharing, harmonization, and reuse across multiple COVID-19 research programs and data sources.

**Objective:**

We aim to present the design, implementation, and capabilities of the RADx Data Hub, a cloud-based platform developed to support findable, accessible, interoperable, reusable (FAIR) data practices and enable secondary analyses of the COVID-19–related data contributed by a nationwide network of researchers.

**Methods:**

The RADx Data Hub was developed on a scalable cloud infrastructure, grounded in the FAIR data principles. The platform integrates heterogeneous data types—including clinical data, diagnostic test results, behavioral data, and social determinants of health—submitted by over 100 research organizations across 46 US states and territories. The data pipeline includes automated and manual processes for deidentification, quality validation, expert curation, and harmonization. Metadata standards are enforced using tools such as the Center for Expanded Data Annotation and Retrieval (CEDAR) Workbench and BioPortal. Data files are structured using a unified specification to support consistent representation and machine-actionable metadata.

**Results:**

As of May 2025, the RADx Data Hub hosts 187 studies and over 1700 data files, spanning 4 RADx programs: RADx Underserved Populations (RADx-UP), RADx Radical (RADx-rad), RADx Tech, and RADx Digital Health Technologies (RADx DHT). The Study Explorer and Analytics Workbench components enable researchers to discover relevant studies, inspect rich metadata, and conduct analyses within a secure cloud-based environment. Harmonized data conforming to a core set of common data elements facilitate cross-study integration and support secondary use. The platform provides persistent identifiers (digital object identifiers) for each study and supports access to structured metadata that adhere to the CEDAR specification, available in both JSON and YAML formats for seamless integration into computational workflows.

**Conclusions:**

The RADx Data Hub successfully addresses key data integration challenges by providing a centralized, FAIR-compliant platform for public health research. Its adaptable architecture and data management practices are designed to support secondary analyses and can be repurposed for other scientific disciplines, strengthening data infrastructure and enhancing preparedness for future health crises.

## Introduction

### Background

The COVID-19 pandemic exposed significant limitations in the data infrastructures needed to support effective public health responses. Fragmented and noninteroperable systems—often operating in silos—hindered the timely collection, integration, and analysis of health data, delaying informed decision-making at both local and global levels [1-3]. As the volume and diversity of pandemic-related data grew, from clinical outcomes and diagnostic testing to genomic surveillance and social determinants, so too did the need for coordinated systems capable of managing, integrating, and analyzing these disparate sources.

In response, a range of national and international initiatives emerged to enable secure, scalable, and structured data sharing. The National COVID Cohort Collaborative in the United States aggregated electronic health records from more than 70 institutions into a centralized enclave for COVID-19 research, applying a common data model to facilitate cross-institutional analyses of clinical outcomes, risk factors, and treatment efficacy [4,5]. The European COVID-19 Data Portal [6], supported by the European Bioinformatics Institute, centralized access to multiomic, epidemiological, and imaging datasets, allowing researchers to explore genomic surveillance trends and viral evolution using standardized pipelines [7]. The ORCHESTRA (Connecting European Cohorts to Increase Common and Effective Response to SARS-CoV-2 Pandemic) project [8] built a federated infrastructure that harmonized data across longitudinal clinical cohorts in Europe, focusing on outcomes among diverse patient populations and informing evidence-based pandemic policy. In the United Kingdom, the OpenSAFELY platform [9] enabled secure, in situ analyses of electronic health records for over 58 million patients, providing rapid insights into COVID-19 risk factors and treatment outcomes. The US Centers for Disease Control and Prevention–led Data Modernization Initiative [10] aimed to transform national public health data infrastructure, catalyzed in part by COVID-19–related gaps in data access, interoperability, and surveillance. In parallel, domain-specific initiatives, such as the Infectious Diseases Data Observatory [11] and Global.health [12] addressed global data equity by supporting data sharing and outbreak analytics in low- and middle-income settings, including resources for contact tracing, genomic epidemiology, and real-time surveillance.

Despite these advances, implementations varied widely in scope, data quality, and adherence to data governance best practices. Many data-sharing efforts were created reactively, often without sustainable frameworks for data stewardship or unified governance mechanisms. While several platforms implemented elements of the findable, accessible, interoperable, reusable (FAIR) principles, such as assigning persistent identifiers or using standardized vocabularies, compliance was inconsistent and frequently deprioritized due to operational urgency [13]. The absence of common data models, interoperable metadata schemas, and cross-platform compatibility limited the potential for integrated analyses across datasets. Moreover, key aspects of reusability—such as provenance tracking, licensing, and semantic alignment—were often underdeveloped or absent altogether. These limitations led to duplication of effort, inefficient data discovery, and barriers to secondary analyses, particularly in domains that required rapid hypothesis testing and multicohort validation, such as diagnostics and health disparities research.

These challenges highlighted the need for a more coordinated and principled approach to data infrastructure—one that could enable consistent metadata standards, facilitate integration across studies, and support responsible data reuse at scale. To address gaps in data standardization and the lack of a centralized platform, the US National Institutes of Health (NIH) launched the Rapid Acceleration of Diagnostics (RADx) initiative in 2020 [14], with a key objective to develop such a centralized platform—the RADx Data Hub [15]. This platform aims to aggregate, harmonize, and provide access to data generated by various programs in the overall RADx initiative, enabling data reuse for secondary analyses and facilitating cross-study comparisons. The RADx Data Hub supports innovative research into diagnostic tools, implementation strategies, and health disparities.

At the core of the RADx Data Hub (hereafter referred to as the Data Hub) is a cloud-based repository grounded in the FAIR guiding principles for data sharing [16]. The machinery and the specifications of the Data Hub are designed to make data (1) findable, through curation, annotation, and support for advanced search and data exploration; (2) accessible, through established privacy and security protocols meant to protect sensitive data while supporting data reuse; (3) interoperable, by leveraging semantic technologies and by defining standard data and metadata models for Data Hub entities; and (4) reusable, by developing routines to validate data quality, techniques to harmonize heterogeneous data, and cloud-based infrastructure to support integrated data analysis on sensitive data.

While the Data Hub was developed specifically to host COVID-19 studies, its underlying technologies were intentionally designed to be repurposed for other domains. The architecture, data and metadata models, software components, and protocols provide a reusable framework that can be adapted to support new systems addressing other areas of scientific research.

### Objectives

We aim to present the Data Hub as a key resource for secondary analysis of COVID-19 data and provide details regarding the data management techniques and software tools developed to bring it to fruition as a reliable and accessible data platform.

## Methods

### Overview

The Data Hub’s approach to supporting data reusability and interoperability is built upon 4 key components: (1) a structured model organizing study data, metadata, and documentation; (2) an end-to-end pipeline for data collection and analysis; (3) a scalable, cloud-based system architecture; and (4) a comprehensive data and metadata harmonization framework. These components are complemented by rigorous data deidentification measures to ensure compliance with established privacy standards, resulting in a flexible foundation that can be adapted for use beyond the COVID-19 context to support a wide range of scientific research domains. The following subsections detail each component and illustrate how they interconnect to form a cohesive, FAIR-compliant infrastructure for scientific data collection, harmonization, and analysis.

### A Model to Organize Study Information

The Data Hub sources its data from 4 (C)DCCs (coordination and data collection centers), each aligned with a distinct NIH RADx program—RADx Radical (RADx-rad), RADx Underserved Populations (RADx-UP), RADx Tech, and RADx Digital Health Technologies (RADx DHT) [17]. The RADx-UP program includes a coordination function, whereas RADx-rad, RADx Tech, and RADx DHT focus solely on data collection. The abbreviation (C)DCCs is used to collectively refer to all of them. The (C)DCCs manage the collection, harmonization, and submission of study data to the Data Hub, supporting the mission and focus of their respective RADx programs. RADx-UP, the largest contributor, focuses on health disparities and community-level data collection from underserved populations. RADx-rad explores experimental (radical) technologies for nontraditional COVID-19 detection and surveillance settings. RADx Tech supports the development and validation of diagnostic technologies. RADx DHT generates digital health data from wearable devices, mobile apps, and real-time monitoring platforms. For RADx DHT studies, the Data Hub hosts rich metadata to enable data discovery and reuse, while the underlying data are stored in the Rapid AI Platform for Innovating Data Science repository [18], which is optimized for handling large-scale digital health data. Collectively, the Data Hub integrates diverse research efforts, enabling comprehensive analyses through harmonized study data and metadata.

**Figure 1:**
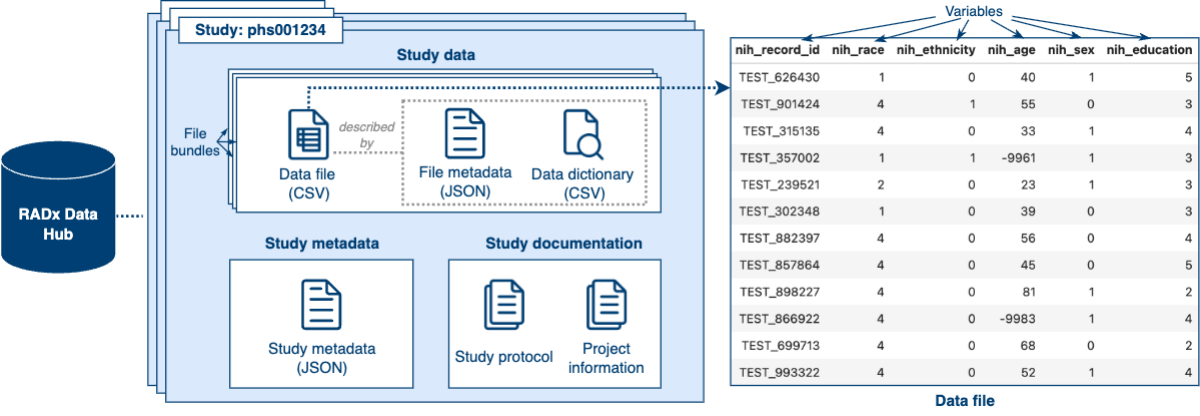
Components of Data Hub studies. This figure illustrates the organization and structure of study data within the Data Hub. On the left, the schematic shows how study data are organized into file bundles, each containing a data file (CSV), file metadata (JSON), and a data dictionary (CSV) that defines the structure and semantics of each variable. Each study is also accompanied by structured study metadata (in JSON format) and supporting study documentation, including protocols and project information. On the right, an example data file is shown, demonstrating a tabular format in which each row corresponds to a study participant and each column represents a standardized variable (eg, race, ethnicity, age, sex, education) harmonized using the Data Hub’s common data elements.

The Data Hub stores information for each study, categorized into 3 overarching components: study data, study metadata, and study documentation (Figure 1). *Study data* constitute the core outputs of each study, capturing the essential variables and results generated during data collection activities. Study data are organized into one or several *file bundles*, each comprising 3 main components (Textbox 1): data file, data dictionary, and file metadata.

**Components of the file bundles.**

**Data file**

A tabular CSV document structured according to its corresponding data dictionary. *Variables*, defined in the data dictionary, represent measurable attributes or characteristics (eg, age, sex, and education) and are organized as columns in the data file. Each row corresponds to a data point, such as an experimental sample or study participant, with values recorded for one or more variables. A data dictionary is essential for interpreting the values in the data file.

**Data dictionary**

A CSV document in which each row unambiguously describes the specification of a variable measured or collected by a study or experiment. The columns of the data dictionary provide identifiers for a variable, including properties such as the variable name, label, unit of measurement, datatype, and other attributes relevant to specifying a variable’s values. The complete specification of data dictionaries used by the Data Hub can be found in the Data Hub Data Dictionary Specification [19]. Data dictionaries are automatically validated against the data dictionary specification upon data submission, and compliance with the specification is strictly enforced. As a result, data dictionaries (and variable metadata, by extension) are amenable to uniform machine processing according to the specification.

**File metadata**

Each data file is paired with a metadata file that describes its key characteristics, including versioning information, creator details, and summary statistics.

Each study is also paired with a *study metadata* file, offering a comprehensive view of its scope, goals, and content. Study metadata includes details such as the study’s geographic and demographic focus, methodologies used, key outcomes, funding sources, and contributing institutions. By providing a consolidated overview, study metadata facilitates the identification and evaluation of studies that are relevant to specific research questions, while enabling systematic reviews and meta-analyses.

In addition, each study is accompanied by *study documentation* that describes the design and execution processes. The study documentation includes study protocols, project summaries, manual of operations, and data-collection instruments. By capturing this rich contextual information, the study documentation helps end users understand the study and use the resulting data effectively. In addition, it offers critical insights into the rationale behind the study design, the measures taken to maintain data quality, and the study’s adherence to regulatory and ethical standards, thereby building trust in the data and facilitating responsible data reuse.

### An End-to-End Pipeline for Data Collection and Analysis

**Figure 2:**
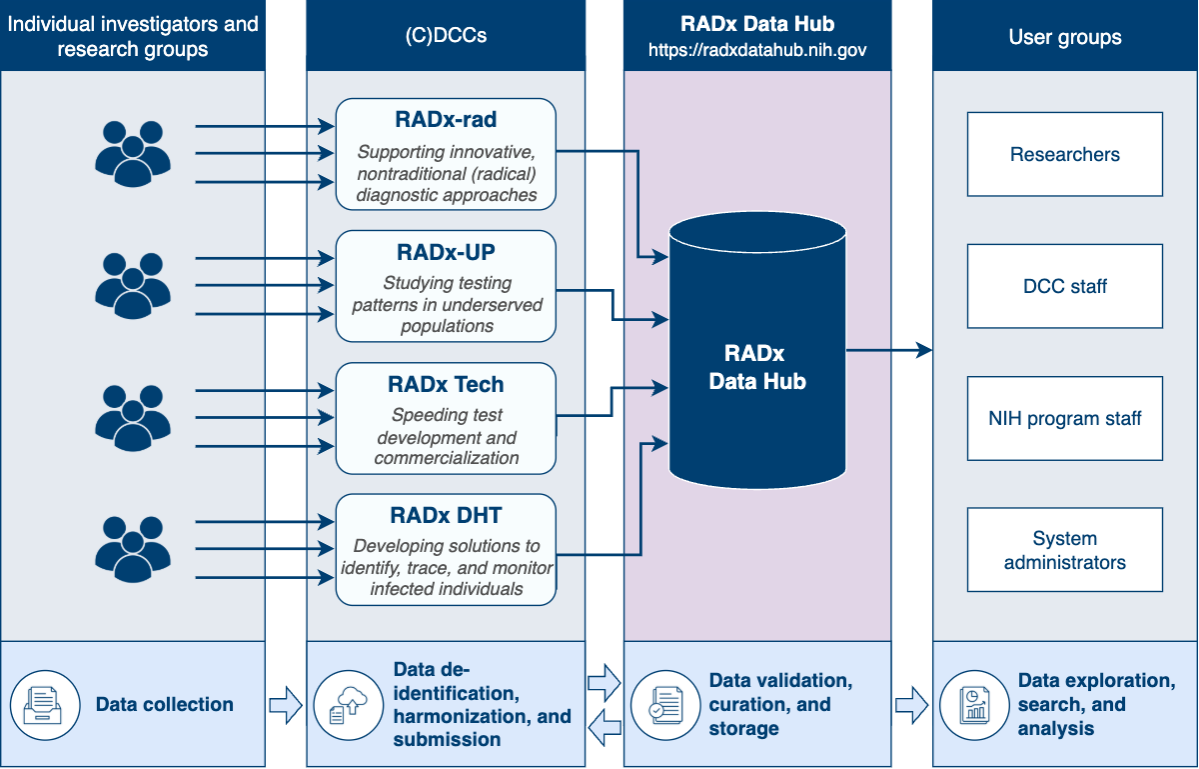
Overview of the Data Hub’s end-to-end data pipeline. Individual investigators and research groups collect and transmit data to the appropriate (C)DCC (coordination and data collection center). At the (C)DCCs, staff deidentify, harmonize, and submit the study data to the Data Hub. Any identified issues are communicated to data contributors, creating a feedback loop for iterative improvements and alignment with Data Hub standards. Once validated, the data are approved, curated, stored in the Data Hub, and then made accessible to users. The arrows reflect the flow of data through the pipeline and are not intended to represent the full range of user interactions with the system.

Data Hub’s end-to-end pipeline (Figure 2) streamlines the collection, deidentification, harmonization, validation, curation, storage, and analysis of the data. Data producers, including individual study investigators and research groups, transmit their study data to one of the 4 (C)DCCs. These (C)DCCs, each aligned with a specific focus within the RADx initiative, play a critical role in the pipeline by standardizing, harmonizing, and preparing the data for inclusion in the Data Hub.

After submission to the Data Hub, the data undergo automated validation to detect quality issues, such as nonadherence to standards, incorrect formats, or incomplete metadata. In addition, the validation process includes automated detection of potential protected health information (PHI) and personally identifiable information (PII) to ensure compliance with privacy and security regulations. These automated steps are followed by manual curation, enabling further refinement of the data. Once these processes are complete, the data are stored and made accessible to users.

#### Overview

The Data Hub leverages the Center for Expanded Data Annotation and Retrieval (CEDAR) Workbench and the BioPortal ontology repository as foundational tools for metadata management and interoperability.

CEDAR [20] offers a suite of web-based tools designed to streamline the creation and management of high-quality metadata. At the core of CEDAR is the concept of metadata templates—structured forms that define the attributes and constraints necessary to describe specific types of data consistently. Templates incorporate controlled vocabularies, specifying permissible values for fields such as demographic characteristics, experimental conditions, and data collection methodologies. Researchers and data contributors use these templates to annotate data with standardized, machine-readable metadata, ensuring adherence to community standards and improving data FAIRness [21].

CEDAR uses the BioPortal ontology repository [22] to integrate ontology terms into metadata templates, ensuring fields are annotated with standardized, semantically rich vocabularies. BioPortal is a comprehensive repository that hosts a vast range of biomedical ontologies, providing standardized terms and definitions across diverse domains to support consistent semantic annotation of data. It provides access to over 1000 ontologies when building CEDAR templates, enabling users to select standardized terms and definitions to describe study variables. By embedding CEDAR’s structured metadata templates and BioPortal’s rich vocabulary resources into its metadata workflow, the Data Hub provides a comprehensive framework for FAIR compliance. The following subsections detail how this framework was used to help ensure the findability, accessibility, interoperability, and reusability of RADx data.

#### Findability: Exploring and Searching Study Data

**Figure 3:**
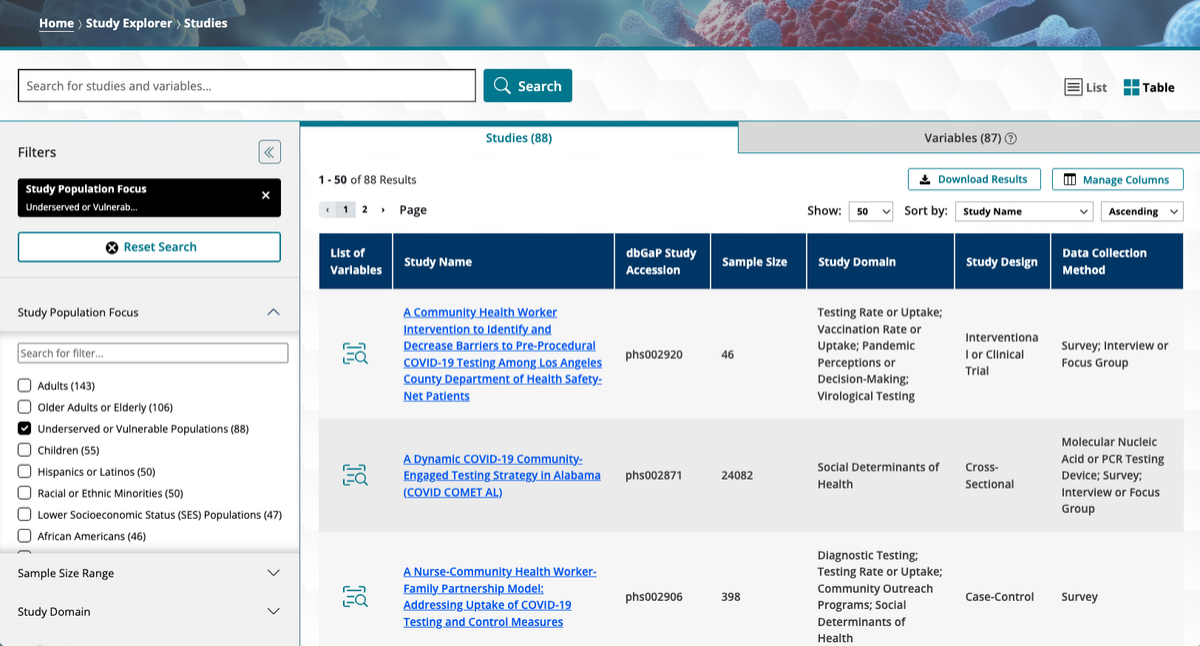
Study Explorer. The figure shows a screenshot of the Data Hub Study Explorer, with the “study population focus” filter applied to retrieve 88 studies on underserved or vulnerable populations. A search box at the top, equipped with an autocomplete feature, allows users to quickly find studies or variables by entering relevant keywords. The interface includes Studies and Variables tabs, enabling users to explore not only study-level metadata but also variable-level details across studies. The filtered results are displayed in a table that includes information such as study names, Database of Genotypes and Phenotypes accessions, sample sizes, study domains, study designs, and data collection methods.

One of the primary purposes of the Data Hub is to provide a platform for researchers to find study data appropriate for their secondary analyses. While access to study data is governed by the NIH Database of Genotypes and Phenotypes (dbGaP) [23] and requires formal approval, the Study Explorer component of the Data Hub (Figure 3) is openly available. It allows users to browse the list of studies, explore their characteristics, and assess their suitability before submitting a data access request through dbGaP. In addition to study-level exploration, the Study Explorer includes a Variables tab that enables users to search across all variables indexed in the Data Hub, supporting discovery of specific data elements of interest across multiple studies. The Study Explorer offers a range of search functionalities, including free-text search, conditional queries using multiple criteria, and options to sort, filter, and download results. Users can filter results using various facets, including study domain, sample size, and data collection method. Studies can also be located directly by querying for their title or other study attributes.

**Figure 4:**
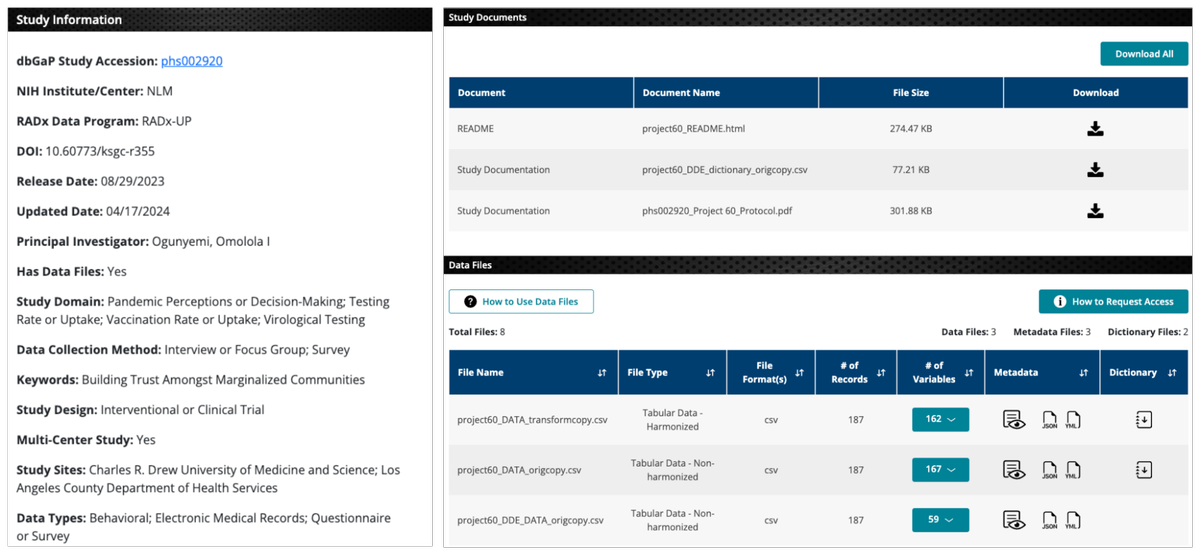
Study Overview page. This figure provides an overview of the information available for a selected study within the Data Hub. The panel at left summarizes key metadata, such as the Database of Genotypes and Phenotypes (dbGaP) study accession, program, study domain, and data collection methods. The panel at right is divided into Study Documents and Data Files. The Study Documents section lists supporting files, such as study protocols and README files. The Data Files section contains the data files and their corresponding metadata, along with a button to request access to the data through the study’s dbGaP page.

**Figure 5:**
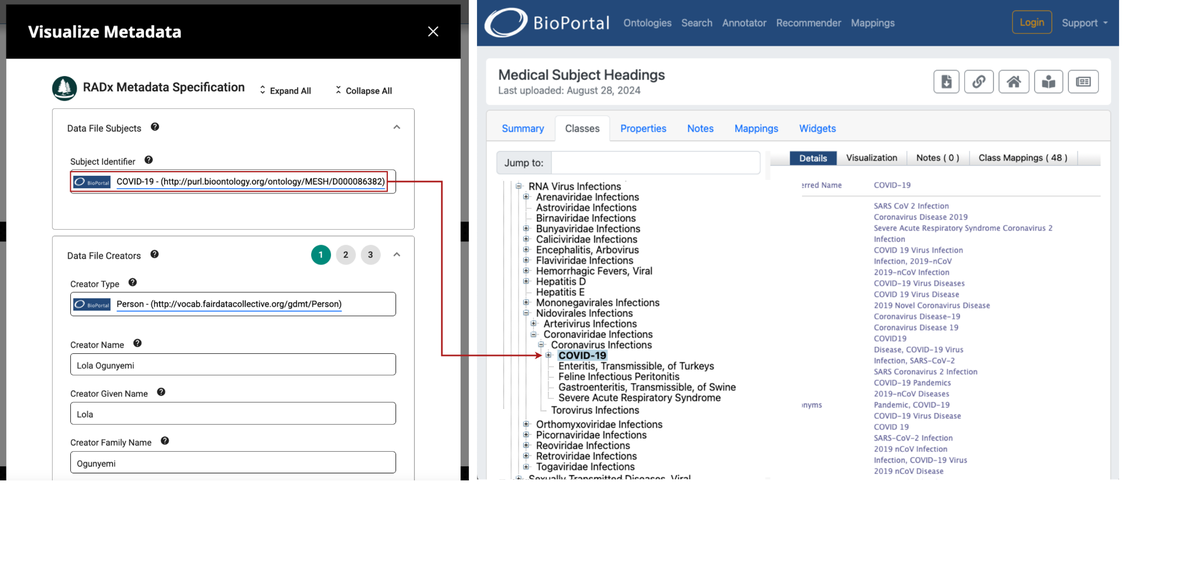
Metadata Viewer. The left side of the figure displays metadata for a specific data file, with terms constrained to ontology terms, linked to BioPortal for further exploration. The right side demonstrates a link from the “subject identifier” field to the corresponding term in the Medical Subject Headings (MeSH). Metadata are organized into expandable and collapsible sections for easy navigation, with help text providing guidance and context. The Metadata Viewer renders a Center for Expanded Data Annotation and Retrieval (CEDAR) template designed for Data Hub files, ensuring consistency and accessibility across metadata records.

Upon locating a study of interest, users can view its details on the Study Overview page (Figure 4). This web page provides a concise summary of the study, offering access to the key components: study metadata, documentation, and data files, including file metadata and data dictionaries. Study metadata can be downloaded in machine-readable formats, such as JSON and YAML, for easy integration into research workflows. To simplify metadata viewing, the Data Hub provides an interactive Metadata Viewer (Figure 5) powered by CEDAR’s Embeddable Editor [24], which includes links to ontology terms from the BioPortal ontology repository [22,25]. This tool allows users to inspect all metadata associated with a study and to verify that the study aligns with their research goals. In addition, the Study Overview page displays the variables used in each file, enabling researchers to identify variables relevant to their analyses or to search for related studies that share the same variables. Furthermore, each study on the Data Hub is assigned a unique DataCite digital object identifier (DOI), a globally persistent identifier that provides a permanent reference to the study and its metadata, enabling reliable citation and access from both within the Data Hub and external sources.

#### Accessibility: Ensuring Data Availability and Secure Access

Protecting sensitive health-related data requires robust control mechanisms to ensure compliance with ethical standards, government regulations, and data use agreements. Access to files from the Data Hub is reviewed and authorized by the NIH Data Access Committee with an established data use agreement through dbGaP [23]. This agreement outlines specific terms for data use, including requirements for privacy protections and ethical conduct. Note that in dbGaP terminology, the document submitted to request access to controlled-access data is formally called a data use certification, which specifies the terms and conditions for data use. The term *data use agreement* is used more broadly across NIH to refer to the framework governing data access. In addition, users must follow the Data Hub user code of conduct [26], which sets standards for responsible data handling and site use.

To formally request access to study-level data, users must log into the Data Hub using their electronic Research Administration Commons or NIH credentials, locate the desired study (or studies), and submit an access request. This requirement ensures that only verified researchers and authorized personnel can access controlled datasets, in compliance with NIH security and data governance policies. While this log-in process may pose a barrier for new users, it helps protect sensitive research data and aligns with NIH’s data access standards. Any individual with principal investigator status can obtain an electronic Research Administration Commons log-in and dbGaP authorization. Once access is approved by the data access committee, principal investigators can manage permissions for their team members via dbGaP. Tutorials on the data request process are available on the Data Hub Resource Center web page [27] to assist users with each step.

Study metadata, summary statistics, and documentation remain openly accessible through the Study Explorer and Study Overview pages. This persistent availability allows users to evaluate the relevance of study data and variables without authentication, supporting transparency, discoverability, and reuse. Although these practices help maintain visibility into study-level details even when data files become unavailable, we acknowledge that a formal policy guaranteeing long-term metadata persistence has not yet been established. As part of our commitment to enhancing FAIRness, we plan to explore mechanisms to ensure long-term metadata retention.

#### Interoperability: Enforcing Data and Metadata Standards

The Data Hub establishes a foundation for interoperability by adopting globally recognized data formats and metadata standards, alongside a standardized set of NIH common data elements (CDEs). CDEs are defined as combinations of standardized questions (variables) paired with an enumerated set of possible responses (values) that are common across multiple studies [28]. Variables in data files are mapped to these CDEs to ensure consistent (ie, harmonized) interpretation across studies.

The CDEs used in the Data Hub are defined in the Data Hub Global Codebook [29]. This listing includes 133 core CDEs that provide a consistent framework for representing essential variables, such as demographic and participant health information, across all RADx studies. These CDEs are organized into 12 categories, including race, ethnicity, age, sex, education, domicile, employment, insurance status, disability status, medical history, symptoms, and health status. The Data Hub team is responsible for performing harmonization to these CDEs where possible by using a harmonization methodology detailed in the Data and Metadata Harmonization section of this paper. However, submitted studies are not limited by these CDEs and may include additional variables collected by the original researchers.

Beyond the provision of CDEs, the Data Hub uses standardized file formats and metadata specifications to further enhance interoperability. Data files are provided in CSV format, organized in a consistent structure where variables are represented as columns and rows as individual data points. While CSV files are not inherently self-descriptive or semantically annotated, their accompanying data dictionaries and metadata files include machine-readable definitions that support automated data interpretation and integration. Data dictionaries follow the RADx Data Dictionary Specification [19], also in CSV format, ensuring uniform interpretation of variables. Metadata for data files and studies are formatted in JSON for linked data, adhering to the CEDAR model specification [30]. In addition, study documentation is provided in standard formats, such as PDF and plain text, ensuring accessibility and compatibility for a wide range of users.

In practice, the (C)DCCs collaborate closely with the Data Hub team to ensure compliance with these global standards. The (C)DCCs conduct the initial harmonization, while the Data Hub team maintains the Global Codebook and performs additional post hoc harmonization as needed before studies are made publicly available. These efforts aim to enhance the interoperability of study data within the RADx ecosystem and with external data repositories and analytical platforms. This comprehensive standardization approach maximizes the data’s utility for research and public health applications.

#### Reusability: Prioritizing Data Quality and Enabling Secondary Data Analysis

The Data Hub promotes study data reusability by focusing on 2 critical aspects: ensuring high data quality and providing an integrated platform for secondary data analysis.

##### Data Quality

To ensure study data utility and reliability, the Data Hub uses a rigorous quality assurance and quality control process. This process begins with close collaboration between the Data Hub team and (C)DCC staff during data submission, ensuring adherence to the data and metadata standards for metadata files, data dictionaries, and documentation described in previous sections. Quality assurance and quality control reviews are performed rigorously before data and metadata are made publicly findable, ensuring compliance with privacy policies, alignment with the Data Hub’s standards, and readiness for harmonization and secondary use.

A key aspect of this process involves evaluating file bundle completeness, identifying and addressing missing values, and standardizing metadata formats to ensure consistency across data files and studies. While the quality of the data reflects the efforts of the original data producers, the Data Hub enhances value by applying harmonization practices during ingestion and by documenting any data and metadata gaps or limitations. These enhancements improve transparency, facilitate cross-study comparisons, and make the data more reliable for downstream analyses.

##### Data Analytics Workbench

**Figure 6:**
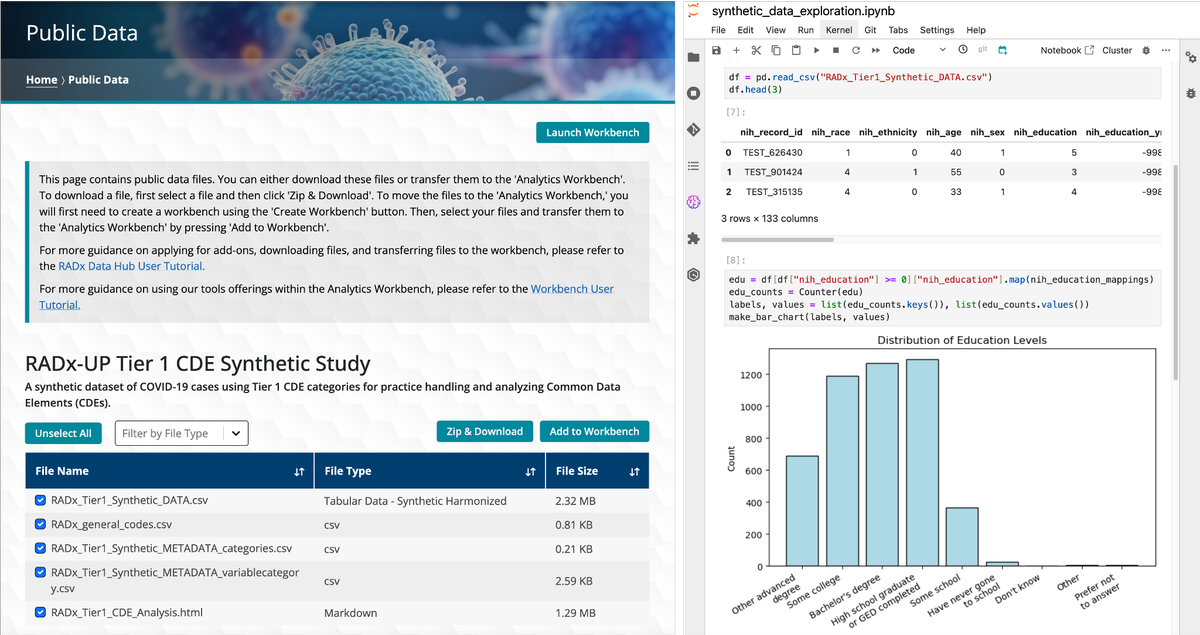
Public data and Analytics Workbench. This figure demonstrates 2 key components of the Data Hub that support data accessibility and exploratory analysis. The left panel shows the Public Data page, where users can browse and download openly available, harmonized synthetic datasets or transfer them to the Analytics Workbench environment. Example files include harmonized data tables, codebooks, and metadata files. The right panel displays the Analytics Workbench in use, where a Jupyter Notebook is used to analyze education-level distributions using bar chart visualizations. This interface supports interactive, code-based data exploration using Python, R, and other tools, enabling secure, scalable, and reproducible analysis directly within the cloud platform.

The Data Hub features a cloud-enabled platform called the Analytics Workbench as a key feature for promoting data reuse (Figure 6). This workbench is a versatile platform, powered by the Amazon Web Services (AWS) SageMaker Studio technology, that enables users to create personalized workspaces and to deploy advanced analytical tools. By leveraging this platform, researchers can securely access, manipulate, and analyze Data Hub data. The workbench provides users with standard data analysis tools, such as RStudio, Python, and JupyterLab, all readily accessible without additional setup. The workbench also supports integration with external platforms, such as DockerHub and GitHub, enabling users to tailor their workflows to specific research needs.

The Public Data page provides access to synthetic data files, which are openly available to users for exploration, testing, and familiarization with the platform’s tools. Users can download these data files directly or transfer them to the Analytics Workbench for further analysis. This feature allows researchers to explore the platform’s tools and capabilities before requesting access to protected study data. The Analytics Workbench is shown on the right-hand side of Figure 6, where a Jupyter Notebook environment allows analysis of the distribution of the individual’s education levels within a synthetic data file. This example highlights the Workbench’s capacity to facilitate secure data analysis and visualization, offering a flexible environment equipped with various analytical tools.

#### FAIR Evaluation

To assess the RADx Data Hub’s adherence to the FAIR principles, we conducted an internal evaluation using the Research Data Alliance (RDA) FAIR data maturity model [31]. This model defines a structured set of indicators and scoring criteria for evaluating findability, accessibility, interoperability, and reusability. The assessment was carried out by members of the Data Hub team with expertise in metadata standards and data governance. Each indicator was evaluated based on available system features and documentation. A summary of the evaluation results is presented in the Results section, with the complete assessment table provided in Multimedia Appendix 1.

### System Architecture

**Figure 7:**
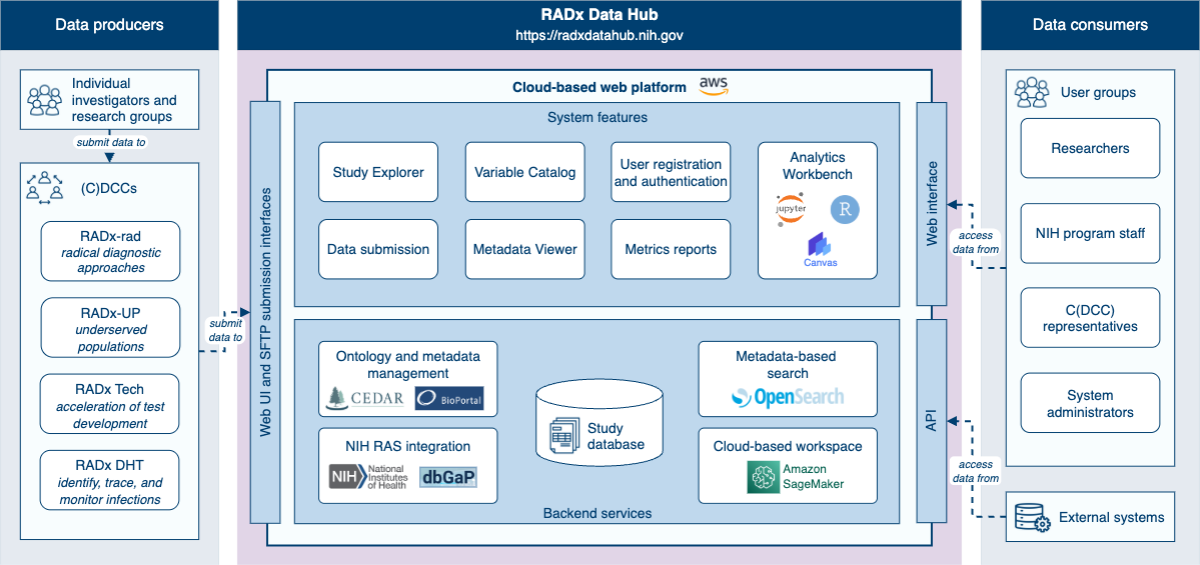
Overview of the Data Hub’s software architecture. This diagram presents the architecture of the Data Hub, highlighting its integration of backend services, web features, and data access pathways. On the left, data producers—including individual investigators and (C)DCCs (coordination and data collection centers)—submit study data via secure web or secure file transfer protocol interfaces. The core platform provides system features such as the Study Explorer, Variable Catalog, Metadata Viewer, and Analytics Workbench, with user authentication managed via the National Institutes of Health’s Researcher Auth Service (RAS) and data access controlled by the Database of Genotypes and Phenotypes (dbGaP). Backend services support ontology-based metadata management (Center for Expanded Data Annotation and Retrieval [CEDAR], BioPortal), metadata-driven search (OpenSearch), and scalable analytics in a cloud-hosted workspace (Amazon SageMaker). On the right, data consumers—including researchers, National Institutes of Health staff, and system administrators—access harmonized study data through web interfaces or application programming interfaces (APIs) for exploration, analysis, and reuse. The modular architecture supports secure, interoperable, and reproducible digital health research across a wide range of public health applications.

The Data Hub is a cloud-based platform designed to provide a scalable, secure, and FAIR-compliant infrastructure for collecting, harmonizing, and analyzing COVID-19 data. The Data Hub architecture supports the flow of diverse data types from data producers to a centralized data repository, enabling researchers and stakeholders to leverage data effectively. The architecture comprises 2 functional layers—the frontend and backend—both hosted on AWS cloud infrastructure and interconnected through secure application programming interfaces (Figure 7). This architecture is designed to scale with increasing data and user demands, while maintaining high performance and availability.

The frontend layer provides user-facing functionalities through a modern web interface built with React and Next.js. The frontend layer supports dynamic page rendering, device compatibility, and accessibility requirements (including compliance with Section 508 of the Rehabilitation Act [32]). Key features include the Study Explorer, Variables Catalog, and Metadata Viewer, which allow users to browse, search, and explore data and metadata, and access secure user registration and authentication systems. Additional capabilities include the Analytics Workbench, which supports advanced data analysis and visualization through interactive environments such as Jupyter for both R and Python. Complementing these tools, Metrics Reports provide actionable insights into data use, quality, and platform performance, supporting effective system monitoring.

The backend layer is implemented using a microservice architecture powered by Spring Boot and Python. It handles business logic and workflows essential for data ingestion, harmonization, and search. Core components include (1) submission services for validating and processing incoming data, (2) search services powered by AWS OpenSearch for efficient metadata-based retrieval, and (3) an integrated analytics workbench leveraging AWS SageMaker for scalable machine learning workflows. Security and compliance are maintained through robust identity-management systems, including NIH Researcher Auth Service integration and role-based access control.

The system’s software architecture is designed for flexibility and scalability to accommodate additional COVID-19 study data, facilitate the addition of new features, and support the potential expansion into other health domains. A Postgres relational database, managed via the AWS Relational Database Service, stores metadata, while raw data files (without metadata) are housed in Amazon Simple Storage Service. This separation ensures efficient querying and modular scalability. AWS OpenSearch indexes metadata to provide fast and precise search capabilities. CEDAR and BioPortal enable metadata annotation and semantic interoperability, ensuring adherence to FAIR principles.

The cloud infrastructure is built on AWS, leveraging serverless technologies, such as AWS Fargate and Lambda for containerized microservices and tasks, and Elastic Load Balancers for high availability. Development, testing, and production environments are isolated to enhance security and streamline deployment workflows. Automated continuous integration and continuous delivery pipelines enable rapid and reliable updates to the system, maintaining operational stability and performance.

### Data and Metadata Harmonization

Harmonization is a key component of the Data Hub’s mission to standardize study data, enabling cross-study analyses and the re-exploration of data within individual studies. Harmonization can be approached in two ways: (1) *prospective harmonization*, where common data and metadata standards are defined and established ahead of data collection, and (2) *retrospective harmonization*, which enforces common data standards and ensures consistent representation of semantically equivalent data through careful curation and processing after the data have been collected [33-35]. Given the urgent, time-sensitive nature of the RADx initiative, comprehensive prospective harmonization was not feasible. Developing and implementing advanced standards before data collection would have delayed critical public health research efforts. Therefore, retrospective harmonization was adopted as a pragmatic solution, enabling the standardization of diverse information while ensuring timely data availability for the broader goals of the RADx initiative.

**Figure 8:**
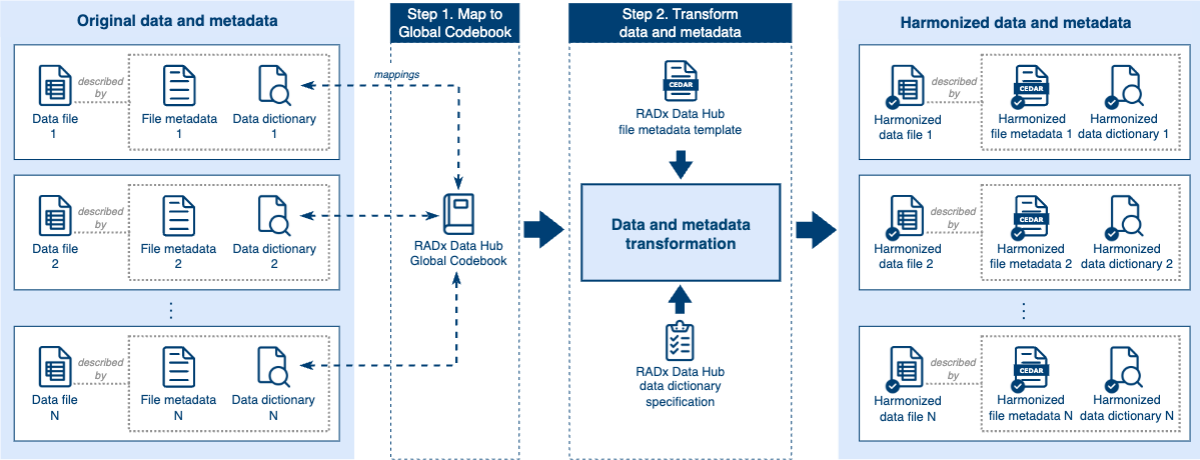
Harmonization workflow for data and metadata. This figure outlines the two-step process used to harmonize incoming data files and metadata within the Data Hub. The Data Hub receives original data files, accompanied by their respective metadata files and data dictionaries (left). In step 1, the original data dictionaries are mapped to the Data Hub Global Codebook for common data elements, standardizing variable definitions and allowable values across studies. In step 2, the original data and metadata are transformed into harmonized formats: data files are transformed using mappings from step 1; metadata files are aligned to a Center for Expanded Data Annotation and Retrieval (CEDAR) metadata template; and data dictionaries are converted to the Data Hub Data Dictionary Specification (center). This process ensures the resulting harmonized data files, metadata, and dictionaries are consistent and interoperable across the Data Hub (right).

The harmonization process within the Data Hub (Figure 8), involves two main steps: (1) mapping data dictionaries to the Data Hub’s Global Codebook [29] and (2) transforming data and metadata to align with standardized templates.

The harmonization workflow begins with mapping original data dictionaries provided by investigators to the CDEs in the Global Codebook, which serve as a unified framework for standardizing variable definitions and values across studies. The identification and creation of these mappings are performed manually by the Data Hub team, leveraging their expertise to account for the variability and complexity of submitted data. The resulting mappings are stored in the Global Codebook, creating a centralized, reproducible resource that supports ongoing and future harmonization efforts.

Following the mapping process, data and metadata are transformed to achieve harmonization, ensuring consistency and interoperability across datasets. Data files are transformed by the (C)DCCs using the mappings identified previously. Study-level metadata (eg, principal investigator information, funding sources, and institutional affiliations) are aligned with a dedicated CEDAR metadata template [36]. Similarly, data file metadata (eg, file name, version, and creator) are structured using a CEDAR template [37] inspired by the DataCite Metadata Schema [38]. The DataCite Metadata Schema provides a widely recognized list of standard core metadata properties for citation and retrieval purposes, which the CEDAR template augments by incorporating additional fields tailored to specific needs, such as detailed funding information and file characteristics. By leveraging the strengths of the DataCite standard while extending its functionality, the CEDAR template ensures that metadata are both globally compatible and capable of meeting diverse research requirements. In addition, data dictionaries are converted to the Data Hub Data Dictionary Specification [19], ensuring uniformity in structure and representation.

**Figure 9:**
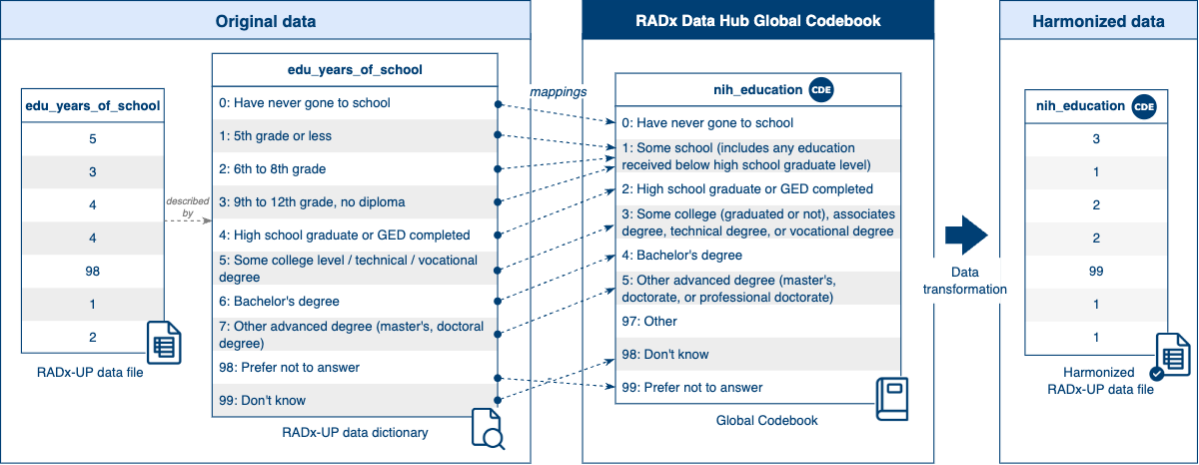
Data harmonization example. This figure illustrates the transformation of an original variable, "edu_years_of_school," from a RADx-UP study into a harmonized common data element (CDE) named nih_education using the Data Hub’s Global Codebook. The left panel displays original data values and their definitions as captured in the study-specific data dictionary. In the center, these original values are mapped to standardized values in the Global Codebook, aligning semantically equivalent categories across studies. For instance, “6: Bachelor’s degree” in the original dictionary maps to “4: Bachelor’s degree” in the CDE. The right panel shows the resulting harmonized data, where the variable name and values have been transformed to conform with the CDE definition, ensuring consistency and interoperability for cross-study analyses.

Figure 9 illustrates an example of data harmonization for the variable *edu_years_of_school*, found in an original RADx-UP data file, representing a study participant’s level of education. On the left, the original variable includes values and corresponding definitions outlined in the RADx-UP data dictionary. For example, a value of *3* represents *9th to 12th grade, no diploma*, whereas *4* corresponds to *High school graduate or GED completed*.

The center panel demonstrates how these original values and definitions are mapped to the corresponding CDE, *nih_education*, within the Data Hub Global Codebook. For instance, the value *4* in the original data file maps to *2: High school graduate or GED completed* in the Global Codebook, ensuring semantic consistency.

Finally, the right panel shows the harmonized data file, where the variable has been renamed *nih_education* and the values updated according to the mappings. This harmonized format ensures that the data are consistent and interoperable within the Data Hub, facilitating cross-study analysis and integration.

The harmonization process is supported by rigorous quality assurance measures conducted by the Data Hub team. Automated tools and structured workflows check for compliance with CDE standards, formatting specifications, and metadata guidelines. Any discrepancies or issues are communicated back to data contributors, creating a feedback loop that promotes iterative improvements and alignment with the Data Hub’s standards (Figure 2). This quality assurance process ensures that harmonized data are reliable, consistent, and ready for reuse.

To ensure semantic consistency across studies, harmonization mappings to CDEs are collaboratively developed by domain experts from both the (C)DCCs and the Data Hub team. Each mapping undergoes manual review to validate alignment with the Data Hub Global Codebook, ensuring standardized interpretation of variables and values. This dual-review approach provides iterative feedback and consensus-building to improve consistency and reproducibility. Future enhancements may include structured auditing protocols or interrater agreement assessments to further strengthen the harmonization quality.

The Data Hub stores both original and harmonized versions of data files. Harmonized data are recommended for cross-study analyses and data re-exploration due to their standardized format and semantic consistency. However, the original raw data are also retained and made accessible for users requiring unmodified data for specialized research needs. This dual offering supports diverse research applications while ensuring the integrity and reusability of the Data Hub’s data resources.

### Data Deidentification

To protect participant privacy, the (C)DCCs apply a comprehensive deidentification process [39] to study data before their submission to the Data Hub. This process ensures data are anonymized while preserving their scientific utility for secondary research. The (C)DCCs are tasked with balancing PHI and PII removal with the retention of sufficient data to maintain their research value. The Data Hub leverages Amazon Macie at the time of data submission to automatically detect and flag potential PHI and PII. Amazon Macie uses machine learning and pattern matching to identify sensitive data, allowing (C)DCCs to review and apply additional deidentification steps as needed. This automated screening process helps ensure that all data submitted to the Hub meet privacy and regulatory requirements.

The deidentification process follows a standard operating procedure that incorporates several key techniques. Direct identifiers, such as names and addresses, are redacted entirely to eliminate any risk of reidentification. In compliance with Health Insurance Portability and Accountability Act rules, zip codes are generalized by retaining only the first 3 digits, unless the area’s population is 20,000 or fewer, in which case the zip code is replaced with *000*. Dates are shifted by a consistent interval to obscure precise temporal details while preserving temporal relationships within the data. Sites within studies are anonymized using random codes, and participant ages are altered: individuals aged <1 years are recorded as *0*, aged 21 to 89 years are modified by adding or subtracting 2 years, and aged >90 are top-coded as *90*.

These procedures are meticulously documented by the (C)DCCs to ensure traceability, replicability, and accountability within their centers. The Data Hub does not receive the internal linkage or detailed records used by the (C)DCCs to perform deidentification; it relies on the (C)DCCs’ adherence to the Data Hub’s deidentification guidance and on automated PHI and PII detection at submission time. In addition, each submitted file undergoes a manual and thorough deidentification review by the Data Hub team to ensure the absence of PII or PHI, and files are approved for release only if no issues are identified. By applying these deidentification standards, the Data Hub safeguards participant privacy while enabling meaningful secondary use of the data for public health research.

### Ethical Considerations

The Data Hub hosts data originally collected by NIH-funded studies and submitted to the Data Hub by third-party (C)DCCs. These (C)DCCs gather data from studies conducted by original investigators, each of which obtained an institutional review board (IRB) approval or a documented waiver at the time of primary data collection. The (C)DCCs are responsible for managing participant consent, including documentation of informed consent forms and any applicable IRB waivers. The Data Hub does not collect data directly from participants. It receives only deidentified data that have been processed by the (C)DCCs in accordance with the Data Hub’s deidentification guidance. Only data from participants who consented to the sharing of their deidentified information are submitted. Because the Data Hub handles only deidentified secondary data for which consent and IRB oversight are managed upstream, no additional IRB approval is required for its operation or for most secondary analyses it supports. However, certain studies may include data elements that require IRB approval at the time of data access request, in accordance with the NIH dbGaP policies. Data access and use are governed by a Data Use Certification through dbGaP, ensuring compliance with data protection and ethical standards. Participant compensation, if applicable, is determined and administered by the original study teams in accordance with their IRB-approved protocols and is not processed or tracked by the Data Hub. The platform does not host or disseminate any identifiable personal information, multimedia, or supplementary materials.

## Results

### Overview

As of May 2025, the Data Hub hosts 187 studies and over 1700 data files contributed by researchers from more than 100 organizations across 46 US states and territories. The Data Hub represents one of the largest coordinated efforts to centralize COVID-19-related public health data, supporting both immediate and long-term responses to health crises. By adhering to the FAIR principles, the Data Hub enhances access to high-quality, standardized study data for secondary analyses and public health research.

The studies stored in the Data Hub cover a broad range of domains, including community-based research, diagnostic technologies, and surveillance in underserved populations. Metadata and documentation for these studies are openly accessible through the Study Explorer, enabling researchers to evaluate the relevance of datasets before requesting full access via dbGaP [23]. This governance ensures compliance with privacy protections and ethical standards while facilitating secure access to sensitive health data.

**Figure 10:**
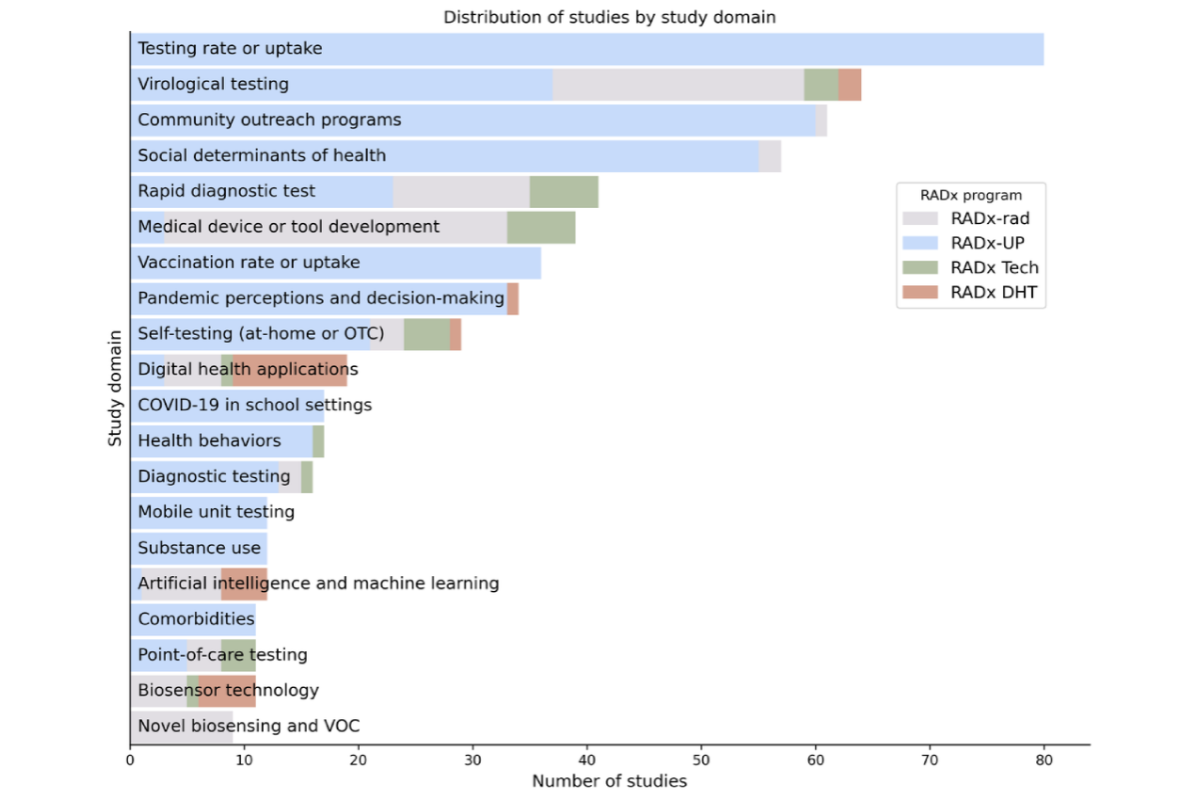
Distribution of Data Hub studies by study domain. This histogram illustrates the distribution of studies across various research domains, highlighting the diverse focus areas of RADx studies. RADx-UP accounts for the largest share of studies, particularly in domains such as community outreach, social determinants of health, and vaccination. The notable representation of RADx-rad in areas like rapid diagnostics and biosensing underscores the emphasis on novel and experimental approaches to COVID-19 detection and monitoring. This distribution highlights the cross-programmatic diversity and strategic focus areas of the RADx initiative. Note: Only the top 20 study domains are shown; additional study domains were excluded for clarity.

**Figure 11:**
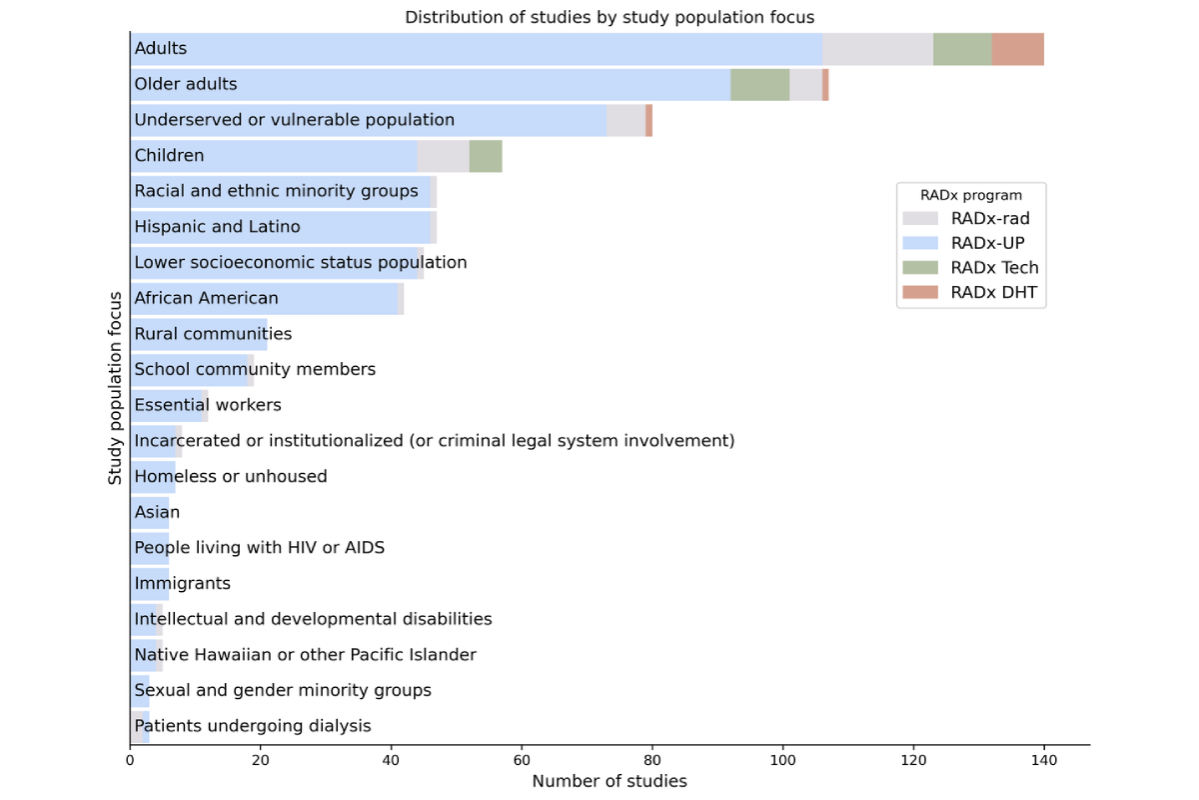
Distribution of Data Hub studies by population focus. This chart presents the breakdown of studies based on population focus. The figure underscores the RADx-UP program’s emphasis on underserved and vulnerable populations, with substantial representation of studies involving older adults, racial and ethnic minorities, low socioeconomic status groups, rural communities, and incarcerated or institutionalized individuals. While many studies target specific at-risk groups—including children, essential workers, and people living with HIV/AIDS—a significant number also focus on general adult populations. This distribution highlights the wide demographic coverage of the Data Hub, supporting diverse and comparative analyses on health disparities, equity, and access in the context of the COVID-19 pandemic. Note: Only the top 20 population focus categories are shown; additional categories were excluded for clarity.

**Figure 12:**
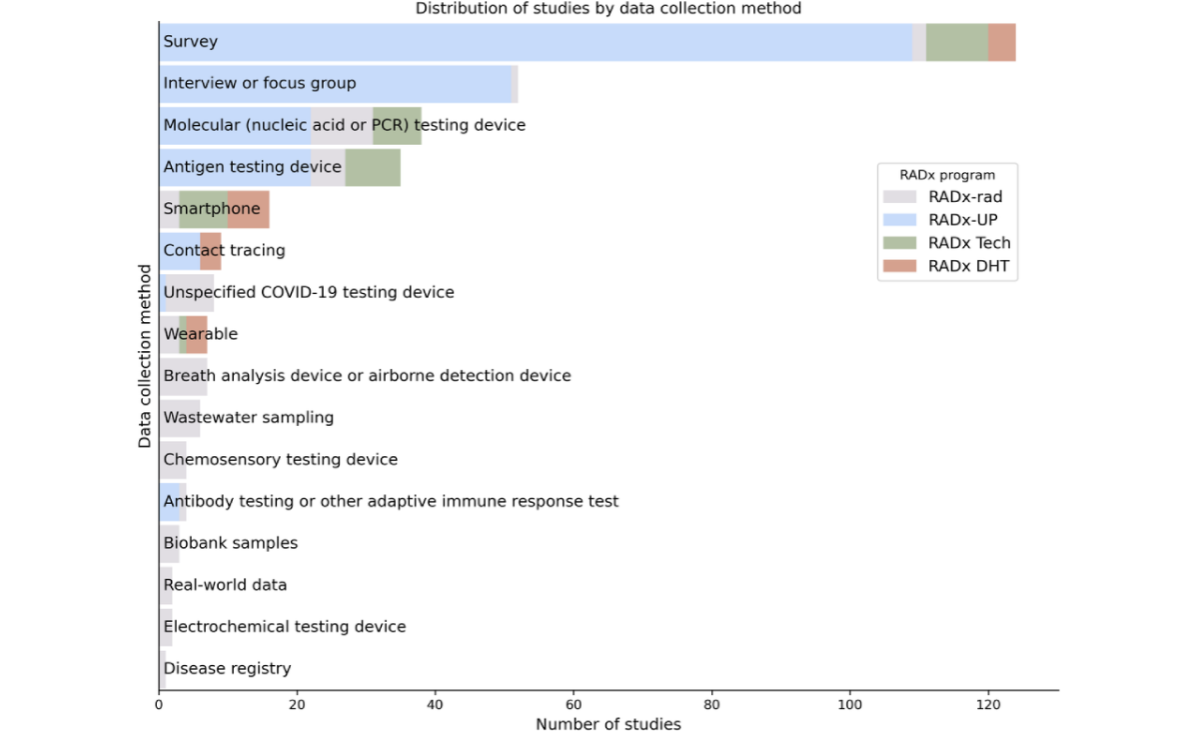
Distribution of Data Hub studies by data collection method. This chart categorizes studies based on their data collection approaches, stratified by RADx program. Survey-based and interview or focus group methods are predominant, particularly among RADx-UP studies, reflecting an emphasis on community engagement and participant-reported outcomes. RADx Tech and RADx DHT programs contribute more heavily to studies utilizing device-based methods, including molecular and antigen testing, wearables, smartphones, and contact tracing tools. Less common but innovative modalities—such as wastewater sampling, breath and chemosensory testing, and electrochemical diagnostics—demonstrate methodological breadth across the RADx portfolio. The heterogeneity of data sources supports integration and cross-study analyses for richer insights into pandemic-related health outcomes.

**Figure 13:**
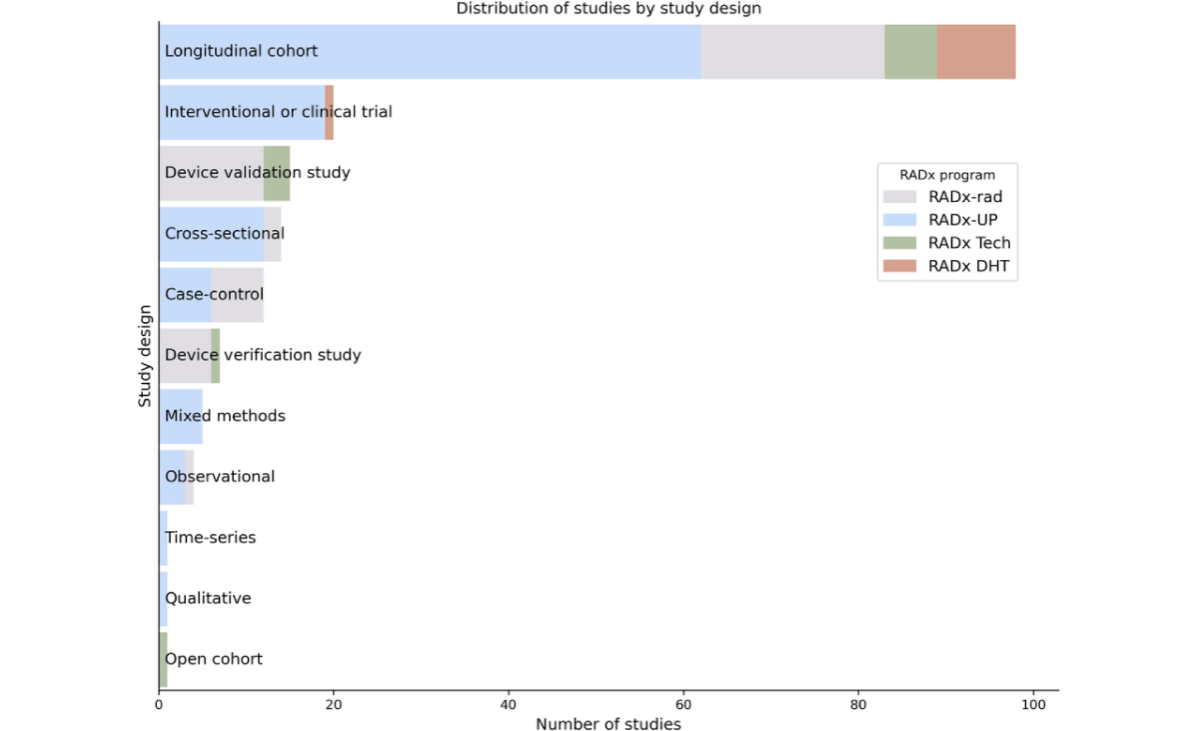
Distribution of Data Hub studies by study design. This chart illustrates the frequency of study designs used across the Data Hub, categorized by program. Longitudinal cohort designs are by far the most common, particularly within RADx-UP, reflecting a focus on long-term, community-based public health research. Interventional or clinical trial designs also feature prominently, especially in RADx Tech and RADx DHT, which emphasize testing and digital health innovation. Additional designs—including cross-sectional, case-control, device validation and verification studies, and mixed methods—demonstrate methodological diversity across the platform. This variety supports a wide range of analyses, from exploratory to evaluative, across observational and experimental frameworks.

Figures 10 to 13 show the distribution of studies by domain, population focus, data collection method, and study design. Stacked bar charts highlight the contributions from the different RADx programs, illustrating the diverse focus areas and methodological approaches across RADx initiatives. The RADx-UP program, with its emphasis on health disparities, contributes a substantial portion of these studies. Many studies focus on underserved populations, including racial minority groups, older adults, and individuals with low socioeconomic status. Data collection methods, such as surveys, wearable device monitoring, and diagnostic testing, offer opportunities for cross-study integration. Most studies follow observational designs.

The Data Hub streamlines data discovery through tools such as the Study Explorer, Variables Catalog, and Metadata Viewer. Each study is assigned a persistent DOI to support long-term access and citation. The platform’s metadata-driven infrastructure enables researchers to efficiently search for and explore studies and variables, reducing barriers to data reuse and collaboration.

The Analytics Workbench offers a secure, cloud-based environment where researchers can access harmonized datasets and perform analyses using tools such as Jupyter and RStudio. By enabling data manipulation, visualization, and advanced analyses directly on the platform, the Workbench reduces local resource demands and improves reproducibility. Researchers can analyze trends, compare study outcomes, and conduct large-scale investigations efficiently without managing their own infrastructure.

The Data Hub’s ability to centralize and harmonize data is crucial in addressing key public health questions related to the COVID-19 pandemic. By enabling multistudy analyses, the Data Hub can facilitate research on health disparities, diagnostic effectiveness, and public health response strategies, reinforcing its role as a crucial resource for pandemic preparedness and response, with significant potential for long-term adaptation to future public health challenges.

The Data Hub’s value is evidenced by the substantial number of peer-reviewed publications derived from the studies it hosts. Since its inception, the RADx initiative has produced over 900 peer-reviewed publications across its constituent programs, addressing topics in diagnostics, health disparities, behavioral science, and digital health. The Data Hub serves as the central repository for the study data underlying many of these publications. Some examples of relevant RADx-supported studies include a qualitative investigation of vaccine hesitancy among staff in skilled nursing facilities [40], environmental surveillance of airborne SARS-CoV-2 particles [41], community-based testing strategies implemented in rural Alabama [42], an epidemiological study on the transition from Delta to Omicron variants [43], modeling responses to pathogens using laboratory-grown placenta models [44], and an analysis of school masking policies and secondary transmission of COVID-19 [45]. These publications exemplify the scientific breadth and translational relevance of RADx-funded research and highlight the Data Hub’s pivotal role in enabling data-driven discovery and public health innovation.

### FAIR Evaluation Results

We evaluated the Data Hub’s alignment with the FAIR principles using the RDA FAIR data maturity model [31], which provides a structured set of indicators across 4 key dimensions: findability, accessibility, interoperability, and reusability. The assessment revealed that the platform demonstrates strong foundational support for FAIR data practices, with several areas for continued improvement. A summary of these findings is provided in Table 1, and a full breakdown of the FAIR maturity assessment is provided in Multimedia Appendix 1.

Findability is supported through the assignment of persistent DOIs to each study. These DOIs resolve to public Study Overview pages that provide descriptive metadata, documentation, and links to related data artifacts. Metadata are structured using CEDAR templates and enriched with ontology terms from BioPortal, enabling semantic search and discovery. The Study Explorer interface further enhances findability by offering keyword search, filtering, and browsing tools.

Accessibility is facilitated through open access to metadata without requiring authentication. Metadata and public data files can be accessed via HTTPS, including support for automated download of synthetic data. For controlled-access data, each Study Overview page links directly to its dbGaP accession, and detailed instructions are provided for users to request access. While authorization is required for sensitive data, access protocols are standardized and widely supported.

Interoperability is enabled by the use of CEDAR templates that incorporate controlled vocabularies, as well as harmonization of many data fields using CDEs from the RADx Global Codebook. Data dictionaries define variable-level metadata and enable machine processing. These practices support semantic consistency across data files and improve the platform’s capacity for integration and automated interpretation.

Reusability is supported through study overview pages that include data use limitations and metadata structured using CEDAR templates for machine readability. Provenance is tracked with the provenance, authoring, and versioning ontology, and metadata files include file-level details, such as contributor information, creation dates, and versioning. Harmonization to CDEs promotes consistent representation of core variables across studies.

**Table 1:** Summary of Rapid Acceleration of Diagnostics (RADx) Data Hub’s adherence to the findable, accessible, interoperable, reusable (FAIR) principles based on the Research Data Alliance (RDA) FAIR data maturity model.

FAIR principle	Strengths	Areas for improvement
Findable	Each study is assigned a DOI^a^ that resolves to a study overview page; detailed and structured metadata are available in machine-readable formats; easy-to-use search and filter tools	No persistent IDs for individual metadata or data records; some metadata lack direct links to data files
Accessible	Metadata include human-readable access information and are manually accessible without log-in; data can be manually accessed via dbGaP^b^ or direct download for public data; study DOIs resolve to overview pages; metadata and data are accessed via HTTPS; public data support automated HTTPS access; metadata remain accessible even if data are not	Metadata lack machine-actionable access information; individual metadata and data files lack persistent IDs; no API^c^ or token-based system for automated access to restricted data; no policy for long-term metadata availability
Interoperable	Metadata are partially annotated with controlled terms from BioPortal; data dictionaries enable machine processing; data fields are harmonized using RADx CDEs^d^	Some metadata fields still use free text and lack URIs^e^; CDEs do not cover all data fields; metadata cross-referencing is still under development; data files lack links to related data or metadata
Reusable	Each study overview page includes data use limitations; provenance is tracked using PAV^f^ ontology; metadata follows CEDAR^g^ specifications for machine readability; data use RADx standards and CDEs	Broader provenance standards and more controlled vocabularies are needed to improve machine understanding

^a^DOI: digital object identifier.

^b^dbGaP: Database of Genotypes and Phenotypes.

^c^API: application programming interface.

^d^CDE: common data element.

^e^URI: uniform resource identifier.

^f^PAV: provenance, authoring, and versioning.

^g^CEDAR: Center for Expanded Data Annotation and Retrieval.

While these features reflect strong FAIR alignment, several areas for improvement remain. For findability, individual data and metadata artifacts—such as specific data files and dictionaries—currently lack persistent identifiers, and DOIs do not resolve directly to stand-alone machine-readable metadata records. In the accessibility dimension, although metadata are openly available, access conditions for restricted data are not yet expressed in machine-actionable formats, and no application programming interface or token-based mechanism is available for automating controlled data access. Regarding interoperability, some key metadata fields, such as study design or domain, still accept free-text values or lack uniform resource identifier–based annotations, and metadata cross-referencing between study- and file-level records is still under development. For reusability, broader provenance standards (eg, PROV Ontology) are not yet implemented, and semantic alignment is incomplete for variables not covered by the Global Codebook.

## Discussion

### Principal Findings

The Data Hub provides a centralized platform to support rapid public health response to the COVID-19 pandemic, overcoming the challenges posed by fragmented public health data systems. By promoting data sharing, reuse, and analysis, the Data Hub enhances data usability and fosters collaboration among researchers, public health officials, and policy makers, ultimately strengthening the ability to respond swiftly and effectively to public health crises, such as the COVID-19 pandemic. The Data Hub uses modern integration techniques and robust data governance practices to ensure data security, privacy protection, and compliance with regulatory standards. The infrastructure and data management techniques developed as part of the Data Hub serve as invaluable tools to combat future public health events by maximizing the utility and impact of shared data within the public health and research communities.

Through rich, structured metadata, the Data Hub improves data findability and enables researchers to quickly assess whether the collected data aligns with their research interests and topic areas before initiating access requests. Moreover, the curation and standardization of data throughout the platform is intended to ensure data quality, resolve inconsistencies and heterogeneity where possible, and identify missing information. Standardizing data formats and metadata enhances interoperability and facilitates data integration, enabling the creation of aggregate data files for large-scale cross-study analyses. These capabilities are vital for evaluating and understanding the COVID-19 pandemic response, addressing disparities in intervention outcomes, advancing biomedical technologies such as testing and rapid detection, and preparing for future pandemics [46-48]. Importantly, the Analytics Workbench reduces common infrastructure barriers—such as the need for local high-performance computing resources, complex software installation, and advanced IT support—by providing a secure, cloud-based environment for reproducible analysis. This design enables more equitable access to advanced data analysis capabilities, particularly benefiting researchers in low-resource institutions or regions where such infrastructure may be limited.

Unlike some pandemic data platforms, the Data Hub prioritizes retrospective harmonization and metadata-driven interoperability. Harmonized data are supported by CDEs specified in the Global Codebook, structured metadata templates via CEDAR, and ontology annotations through BioPortal. These components work in concert with automated validation tools and collaborative curation workflows to ensure semantic consistency across studies. By aligning heterogeneous variables, resolving data quality issues, and enabling ready-to-use, interoperable study data, the platform strengthens the reusability and integration of public health data for secondary research.

### Limitations

While the Data Hub has made significant strides in facilitating data sharing and reuse, its development and implementation have highlighted key challenges in managing a large-scale, multisource data platform. Retrospective harmonization remains a major challenge due to the diversity of study types—ranging from human participants to environmental monitoring—which often require extensive curation to align with evolving metadata models and the RADx Global Codebook. Although data stewards assist contributors during submission, the variability in data quality and standardization across studies demands ongoing refinement. Some datasets must be accepted with minimal curation to ensure repository completeness, emphasizing the need for sustained collaboration with (C)DCCs. In addition, while the data access process—governed by dbGaP and the Data Hub’s user code of conduct—supports ethical and legal compliance, it can pose usability barriers. Researchers must evaluate the potential utility of data using only metadata and study overviews before initiating access requests.

Despite strong alignment with many aspects of the FAIR principles, our structured evaluation using the RDA FAIR data maturity model revealed several limitations that we are actively addressing. These include the lack of persistent identifiers for individual data and metadata artifacts, the continued use of free-text fields where standardized vocabularies would improve consistency, and incomplete links from metadata to the associated data files. We also plan to enhance machine-actionability by encoding access conditions in structured, reusable formats. Addressing these gaps is a key focus of our development road map for advancing FAIR compliance. A detailed assessment is provided in Multimedia Appendix 1.

### Conclusions

The Data Hub addresses critical challenges in public health data management by providing a centralized, FAIR-compliant platform that integrates diverse datasets for secondary analyses. Through its scalable architecture, metadata-driven workflows, and robust privacy measures, the Data Hub has become a pivotal resource for facilitating cross-study research. By enabling rapid access to harmonized COVID-19 data, the platform advances research efforts on health disparities, diagnostic tools, and pandemic preparedness. The cloud-based Analytics Workbench further supports secure, reproducible analysis without local infrastructure requirements. Ongoing enhancements include a software framework to automate integration of heterogeneous data [49] and improvements in metadata quality and usability.

Beyond COVID-19, the Hub’s modular design and governance model position it as a template for future public health data infrastructures. Features such as ontology-annotated metadata, Open Researcher and Contributor Identifier [50], and Research Organization Registry [51] integration, and improved metadata visualization are laying the groundwork for a more interoperable and user-friendly research ecosystem. Planned developments—such as a user-friendly data dictionary viewer, artifact-level persistent identifiers, and expanded use of standardized vocabularies and provenance—directly respond to gaps identified in our FAIR evaluation. These efforts aim to ensure that data remain findable, accessible, interoperable, and reusable across evolving needs. By enabling scalable, standards-based data reuse, the RADx Data Hub supports more agile, collaborative, and equitable research—critical for evidence-based responses to current and future public health challenges.

## Appendix

Multimedia Appendix 1 FAIR assessment results for the RADx Data Hub, conducted using the Research Data Alliance’s FAIR Data Maturity Model. This spreadsheet details the platform’s compliance with each FAIR subprinciple, including identified gaps and planned enhancements.

## Abbreviations

(C)DCC: coordination and data collection center
AWS: Amazon Web Services
CEDAR: Center for Expanded Data Annotation and Retrieval
CDE: common data element
dbGaP: Database of Genotypes and Phenotypes
DOI: digital object identifier
FAIR: findable, accessible, interoperable, reusable
IRB: institutional review board
NIH: National Institutes of Health
PHI: protected health information
PII: personally identifiable information
RADx: Rapid Acceleration of Diagnostics
RADx DHT: RADx Digital Health Technologies
RADx-rad: RADx Radical
RADx-UP: RADx Underserved Populations

## Data Availability

The datasets hosted in the Rapid Acceleration of Diagnostics (RADx) Data Hub are not publicly available due to participant privacy protections and data governance requirements but may be accessed by qualified researchers upon reasonable request. Instructions for submitting a data access request are provided on the RADx Data Hub Resource Center web page [27].
